# A Rare Cause of Abdominal and Flank Pain in Children: Nutcracker Syndrome

**DOI:** 10.7759/cureus.16422

**Published:** 2021-07-16

**Authors:** Ankit Agarwal, Florentina Litra, Lori L Barr

**Affiliations:** 1 Pediatrics, Ascension Sacred Heart, University of Florida, Pensacola, USA; 2 Pediatric Radiology, Radiology Associates of Florida, Radiology Partners, Pensacola, USA

**Keywords:** nutcracker phenomenon, nutcracker syndrome, flank pain, abdominal pain, left renal vein compression

## Abstract

The nutcracker phenomenon is characterized by compression of the left renal vein typically between the abdominal aorta and superior mesenteric artery. It is an uncommon and often undiagnosed condition that has the potential to cause a range of symptoms including hematuria and abdominal or flank pain. The term nutcracker syndrome refers to the clinical manifestations of the nutcracker phenomenon. Diagnosis can be made with Doppler ultrasound, computed tomography angiography, magnetic resonance angiography, or venography. Management can range from conservative treatment in the pediatric population due to high spontaneous remission rate to surgical and endovascular interventions. We discuss the case of a previously healthy young female who presented with abdominal pain. Diagnosis of nutcracker syndrome was made based on imaging. The patient was managed conservatively. This case highlights the importance of considering nutcracker syndrome in the differential diagnosis when evaluating patients with abdominal and flank pain.

## Introduction

The nutcracker phenomenon (NCP) is an uncommon condition and refers to the compression of the left renal vein (LRV), usually between the abdominal aorta and the superior mesenteric artery (SMA). This compression leads to narrowing of the aortomesenteric portion and dilatation of the distal portion of the LRV [1]. Varying degree of extrinsic stenosis of the LRV has results ranging from asymptomatic cases to episodes of macroscopic hematuria, orthostatic proteinuria, renovascular hypertension, abdominal pain, flank pain, dyspareunia, dysmenorrhea, varicocele, and fatigue [2]. Term nutcracker syndrome (NCS), also called LRV entrapment syndrome, is reserved for patients who manifest symptoms secondary to this pathology. The exact prevalence is unknown because of the lack of standard diagnostic criteria and variability of symptoms among people with NCS. It usually affects females with prevalence peaking in young and middle-aged adults [3,4]. The absence of intra-abdominal fat in patients with marked weight loss or leanness promotes the development of symptoms by narrowing the space between SMA and the aorta [5].

## Case presentation

A 13-year-old previously healthy female with a body mass index of 19.1 kg/m^2^, presented with a four-day history of intermittent left-sided flank and abdominal pain. The patient described the pain as dull and aching at rest but exacerbated by movement. She endorsed nausea when the pain was particularly severe but denied emesis. The pain was partly controlled on nonsteroidal anti-inflammatory drugs. The patient denied recent illness, fever, hematuria, dysuria, constipation, or recent trauma to the abdomen. There was no family history of renal disease, diabetes, or hematuria in the family.

**Figure 1:**
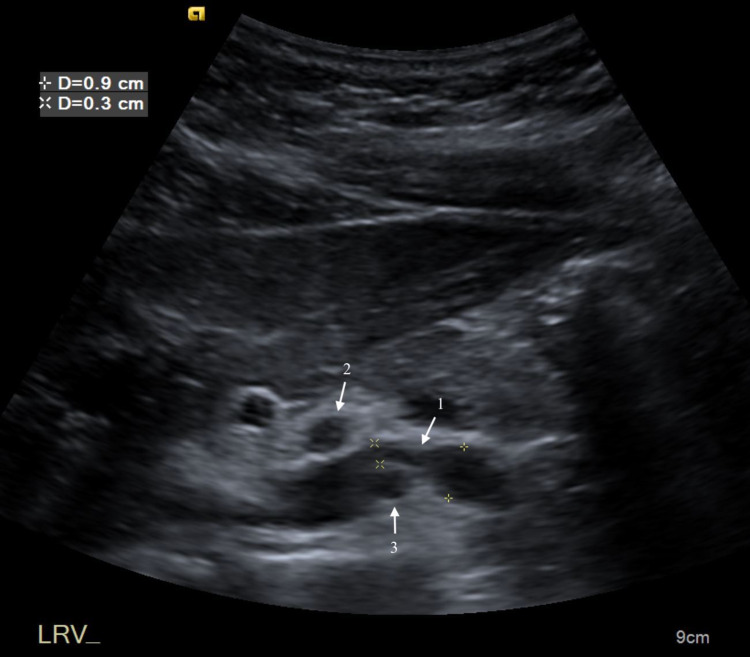
Transverse ultrasound of upper abdomen demonstrates compression of the LRV (1) between SMA (2) and the aorta (3). LRV - left renal vein; SMA - superior mesenteric artery

Physical examination revealed a lean female with mild left flank tenderness on palpation with no rebound or guarding. The pain was slightly exacerbated by trunk flexion and extension. There was no palpable abdominal mass or organomegaly. Respiratory and cardiovascular examinations were unremarkable. Complete blood count and comprehensive metabolic panel were within normal limits. Urinalysis was unremarkable, notably without hematuria, proteinuria, or findings suggestive of urinary tract infection. The pregnancy test was negative. Amylase and lipase to assess for pancreatic pathology were within normal limits. Ultrasound (US) revealed compression of the LRV between SMA and the aorta (Figure 1).

**Figure 2:**
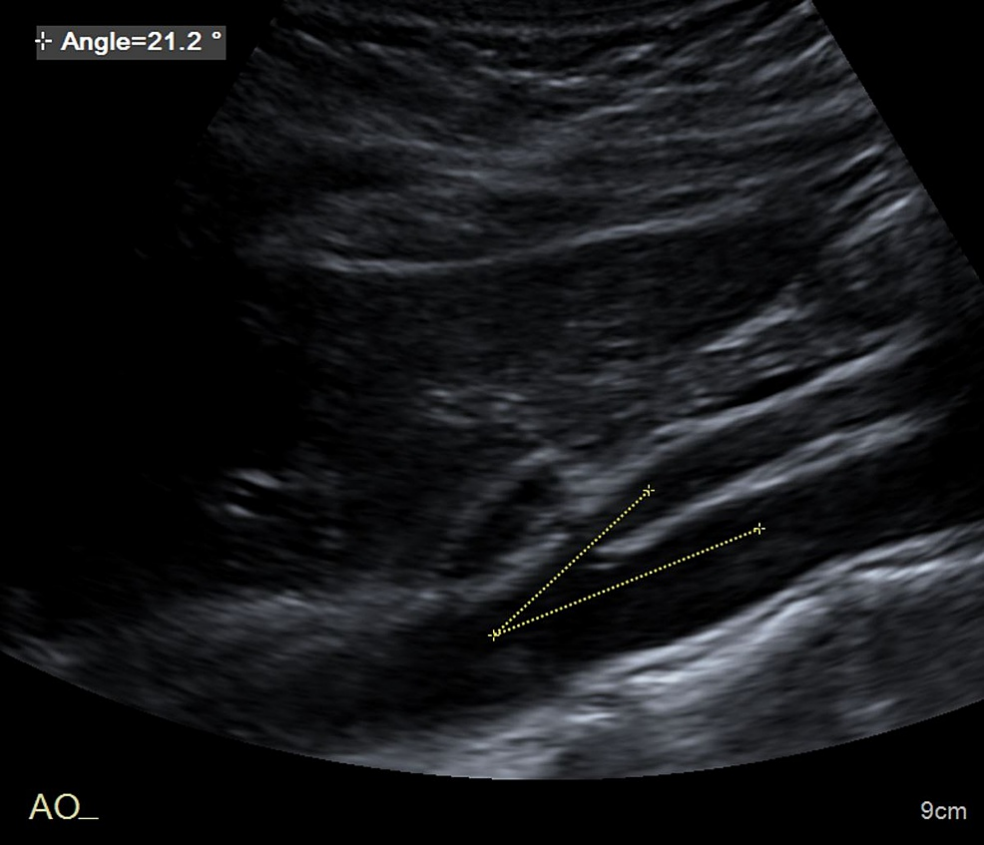
Sagittal abdominal ultrasound shows the angle between SMA and the aorta. SMA - superior mesenteric artery

The anteroposterior (AP) dimension of the LRV decreased to 2 mm posterior to the SMA from 7 mm in the segment proximal to the SMA. There was aliasing of the LRV Doppler waveform at the site of extrinsic compression from the SMA. While velocities in the proximal and distal segments were normal, the velocity diminished to less than 10 cm/s at the site of narrowing. The aorta to SMA angle was determined to be 21.2 degrees (Figure 2).

**Figure 3:**
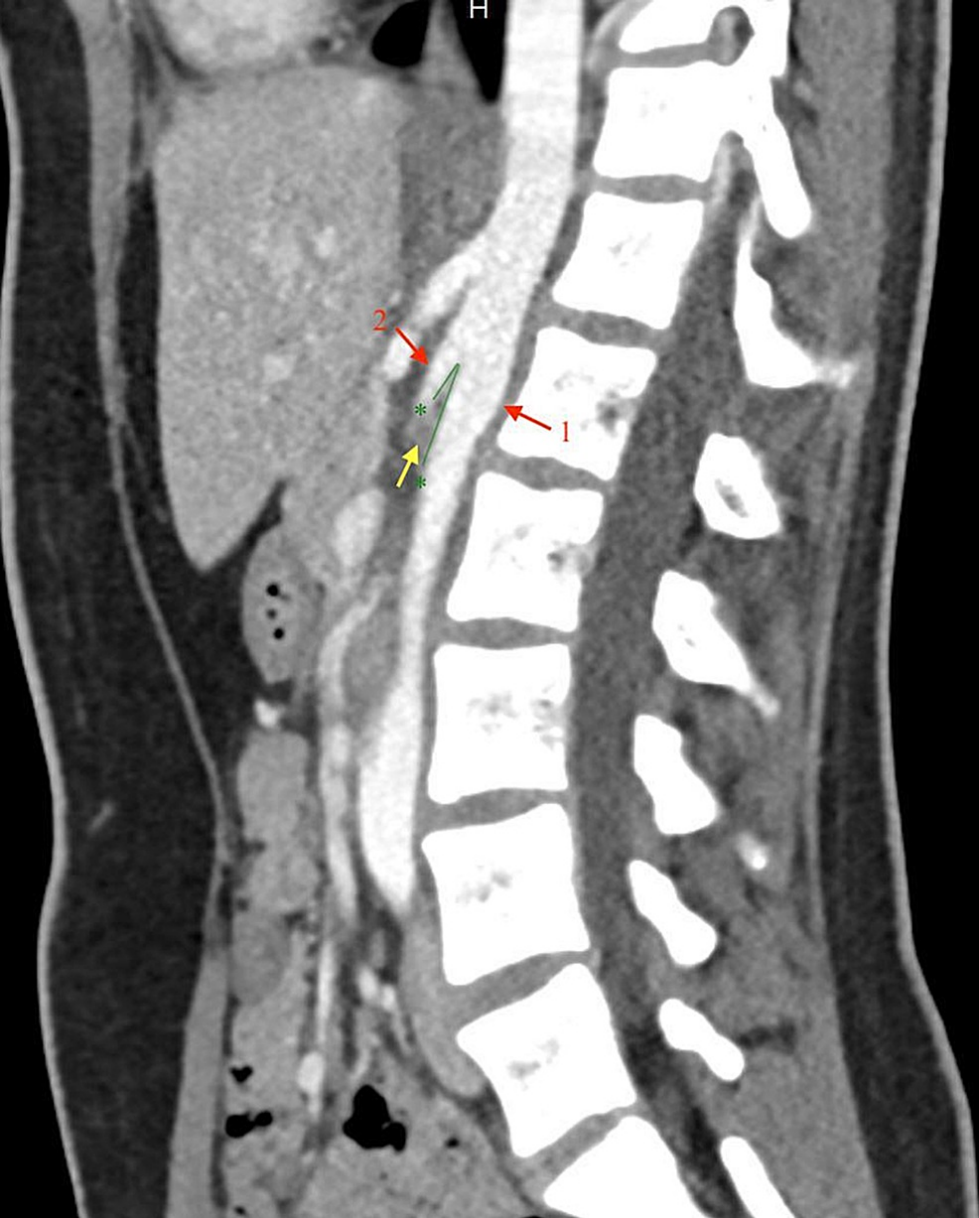
Sagittal CECT shows the aortomesenteric angle (green lines) of 11 degrees. Note resultant compression of the LRV (yellow arrow) between the aorta (1) and the SMA (2). CECT - Contrast-enhanced computed tomography; LRV - left renal vein; SMA - superior mesenteric artery

Contrast-enhanced computed tomography (CECT) of the abdomen and pelvis showed compression of the LRV as it crossed posterior to the SMA and anterior to the aorta. The angle between the aorta and the SMA was 11 degrees (Figure 3).

**Figure 4:**
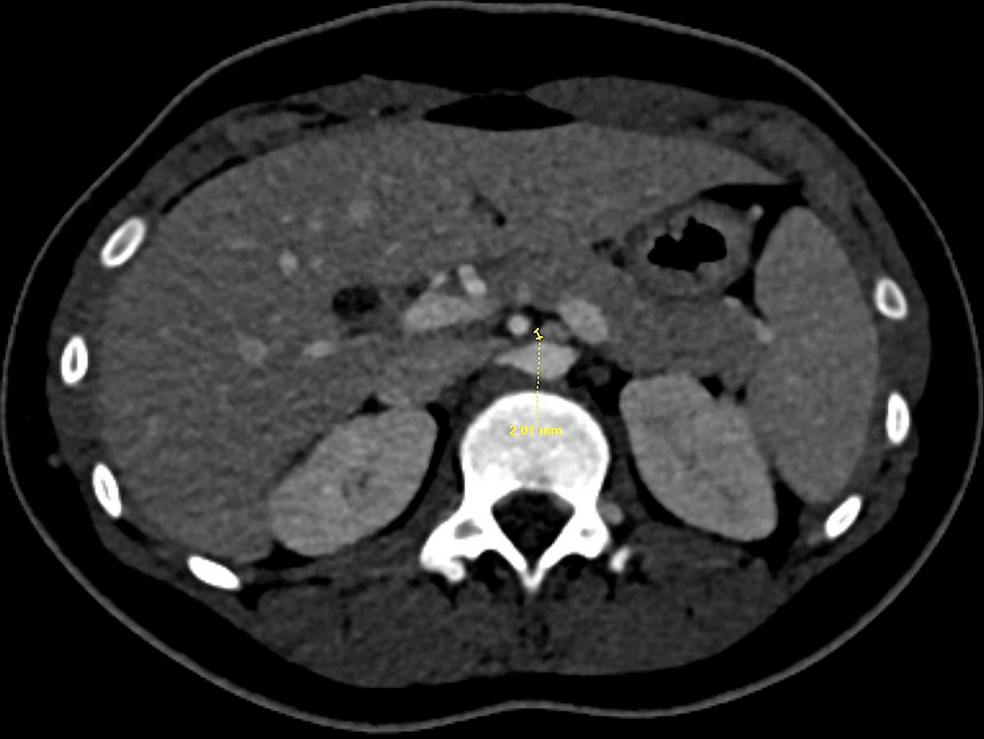
Axial CECT shows a 2-mm AP diameter of the LRV as it crosses between the aorta and SMA. CECT - Contrast-enhanced computed tomography; AP - anteroposterior; LRV - left renal vein; SMA - superior mesenteric artery

**Figure 5:**
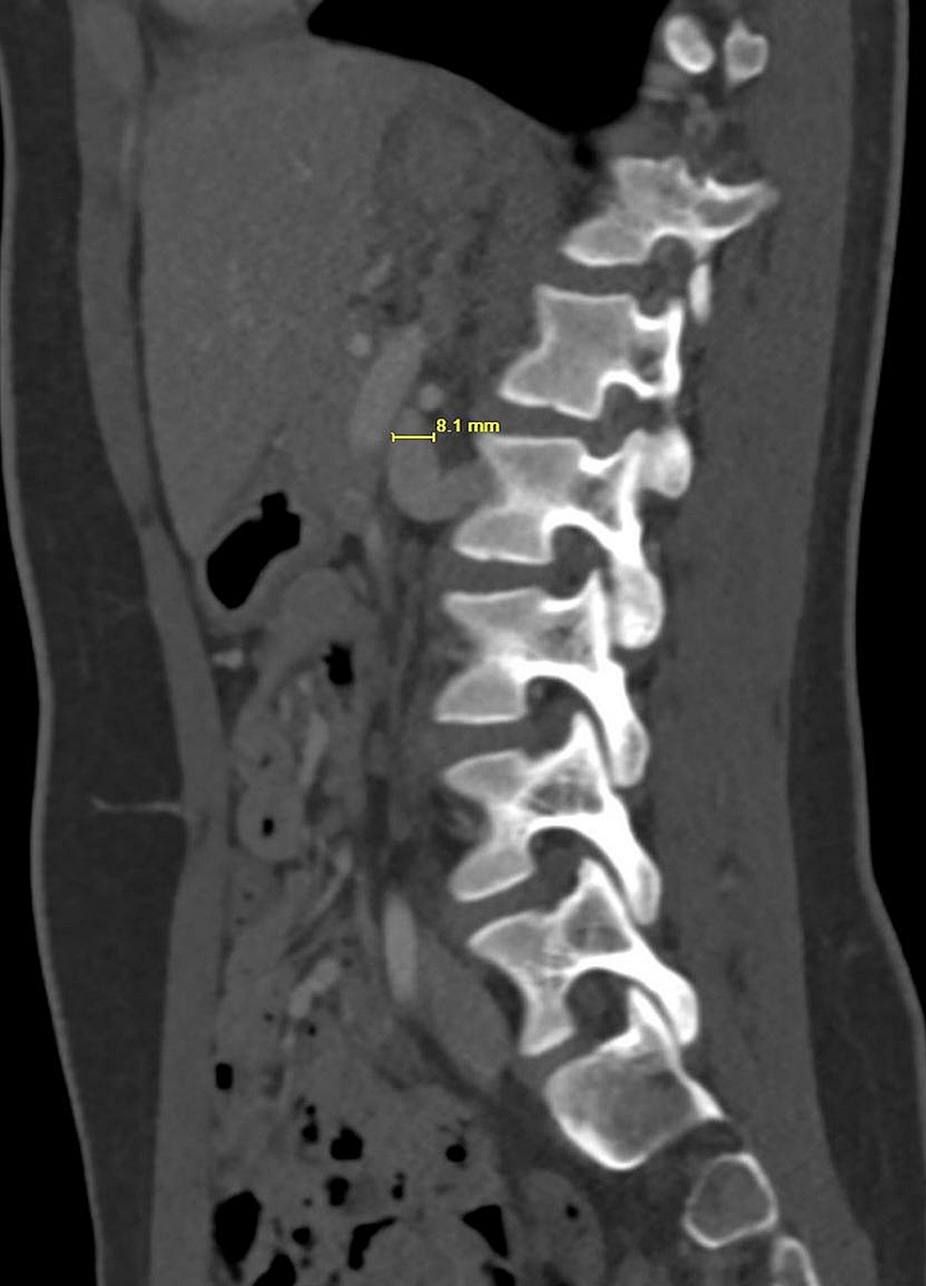
Left parasagittal CECT shows dilatation of the LRV proximal to the compression with an AP diameter of 8 mm. CECT - Contrast-enhanced computed tomography; AP - anteroposterior; LRV - left renal vein

The maximum AP diameter of the LRV posterior to the SMA was only 2 mm whereas 8 mm to the left of the midline (Figures 4, 5).

A prominent left paraspinal vein was seen adjacent to the left side of the LRV suggesting diversion of flow to the left paraspinal venous system. The gonadal vein was not enlarged.

After a multidisciplinary discussion, it was determined that patient was suitable for conservative management and monitoring. She was discharged home to continue outpatient follow-up.

## Discussion

Abdominal and flank pain are common complaints in childhood. A good case history, physical examination, and targeted laboratory investigations can lead to the diagnosis of causes including infection, constipation, renal stones, urinary tract infection, trauma, pyelonephritis, etc. Rare causes like NCS may be missed without a more detailed workup.

Atypical left flank pain occurs in one-third of patients with NCS and is usually explained as referred visceral pain secondary to LRV dilation [6]. High pressure in the compressed LRV results in the formation of venous reflux, venous hypertension, and hence variceal formation between the renal pelvis and ureter. Rupturing of the thin wall dividing the renal collecting system and the small veins results in micro or macrohematuria [7]. Backpressure in the left testicular vein due to LRV compression can lead to a left testicular varicocele. In females, pelvic venous congestion may manifest as dyspareunia, dysmenorrhea, dysuria, and pelvic pain [4]. Patients may not have renal symptoms if well-developed collateral circulation decreasing the LRV hypertension exists.

The presence of the clinical features forms a basis for the diagnosis. The presence of hematuria and proteinuria in a patient must be explored. Urine analysis, urine culture, and imaging of kidneys should be performed. Doppler US (DUS), CT angiography (CTA), magnetic resonance angiography (MRA) and retrograde venography are imaging methods used to diagnose NCS. DUS with sensitivity and specificity as high as 78% and 100%, respectively, is an appropriate initial diagnostic test in patients with suspected NCS [8]. CTA and MRA provide a demonstration of the anatomy and the ability to investigate abdominal findings. These investigations can visualize the precise compression point and the prestenotic dilatation of the LRV as well as gonadal and perirenal vein varices [9]. MRA delineates soft tissue anatomy in the region of compression and has the advantage of less amount of radiation exposure. Retrograde venography with measurement of the venous pressure gradient between the LRV and the inferior vena cava is the gold standard test for the diagnosis of NCS. It is an invasive test and usually unnecessary for the diagnosis [10].

The angle between the aorta and SMA is about 90 degrees, and space is enclosed by lymph nodes, mesenteric fat, and other soft tissues [1]. In NCS, the SMA branches from the aorta at an acute angle causing compression of the LRV. An angle less than 90 degrees is considered significant and is often less than 35 degrees in patients with NCS. Variations in normal anatomy and the lack of stringent criteria make the diagnosis of NCS difficult. US features consistent with NCS are: decreased angle between the aorta and SMA (normal = 38-65), compression of the LRV at the origin of aorta and SMA, increased flow velocity at LRV defect >5:1, left-sided varicocele with a vein lumen diameter of >3 mm [11]. The normal pressure gradient between inferior vena cava and LRV is <1 mmHg. A pressure gradient of >3 mmHg on venography indicates LRV hypertension and is used as a diagnostic criterion for NCS [12]. However, the pressure gradient may be normal due to the development of collateral circulation. Systolic peak velocity of >4.7 between the site of the compression and the vein at the renal hilum on the US is suggestive of NCS [13]. "Beak sign" is the abrupt narrowing of the LRV between the aorta and SMA with proximal dilatation of the LRV. It is considered an accurate CT parameter.

The treatment of NCS is controversial and depends upon the severity of clinical symptoms. The management methods are ranged from observation to nephrectomy. In the pediatric population, conservative treatment is recommended due to the possibility of spontaneous resolution following adipose tissue development. Additionally, the formation of collaterals may further relieve LRV hypertension. Analgesics may be used for pain control. Angiotensin-converting enzyme inhibitors have been shown to improve orthostatic proteinuria associated with NCS [14]. It has been reported that aspirin therapy improves the left-to-right renal perfusion ratio in children with NCS [15]. Surgery should be considered in patients with recurrent gross hematuria with anemia, severe flank pain, kidney dysfunction, or if the symptoms are worsened or not relieved after more than two years of physical therapy [16]. Surgical intervention may be required in the adults if symptoms are severe or continue to exist after six months of conservative therapy. Surgical techniques including open, laparoscopic, and endovascular surgery are performed to decrease the intrarenal venous pressure by releasing the compressed LRV [1].

## Conclusions

NCS is a rare and underdiagnosed phenomenon that has substantial morbidity. Due to the lack of clinical diagnostic criteria, patients undergo multiple procedures and investigations before a diagnosis can be made. Unnecessary investigations and consultations lead to a substantial increase in the financial burden on the patient and the family. NCS should be in the differential diagnoses when evaluating the patients presenting with abdominal and flank pain with other symptoms described. Imaging tests, such as DUS and CTA, should be performed in such patients to confirm the diagnosis.

## References

[1] He Y, Wu Z, Chen S (2014). Nutcracker syndrome--how well do we know it?. Urology.

[2] Novaes LFC, da Silva Saguia LN, Di Migueli CA (2017). Young woman with nutcracker syndrome without main clinic manifestation: hematuria-case report. Int J Surg Case Rep.

[3] Ananthan K, Onida S, Davies AH (2017). Nutcracker syndrome: an update on current diagnostic criteria and management guidelines. Eur J Vasc Endovasc Surg.

[4] Kurklinsky AK, Rooke TW (2010). Nutcracker phenomenon and nutcracker syndrome. Mayo Clin Proc.

[5] Park YB, Lim SH, Ahn JH, Kang E, Myung SC, Shim HJ, Yu SH (2000). Nutcracker syndrome: intravascular stenting approach. Nephrol Dial Transplant.

[6] Alaygut D, Bayram M, Soylu A, Cakmakcı H, Türkmen M, Kavukcu S (2013). Clinical course of children with nutcracker syndrome. Urology.

[7] Stewart BH, Reiman G (1982). Left renal venous hypertension "nutcracker" syndrome. Managed by direct renocaval reimplantation. Urology.

[8] Takebayashi S, Ueki T, Ikeda N, Fujikawa A (1999). Diagnosis of the nutcracker syndrome with color Doppler sonography: correlation with flow patterns on retrograde left renal venography. AJR Am J Roentgenol.

[9] Bhanji A, Malcolm P, Karim M (2010). Nutcracker syndrome and radiographic evaluation of loin pain and hematuria. Am J Kidney Dis.

[10] de Macedo GL, Dos Santos MA, Sarris AB, Gomes RZ (2018). Diagnosis and treatment of the nutcracker syndrome: a review of the last 10 years. J Vasc Bras.

[11] Englund KM, Rayment M (2018). Nutcracker syndrome: a proposed ultrasound protocol. Australasian J Ultrasound Med.

[12] Wolfish NM, McLaine PN, Martin D (1986). Renal vein entrapment syndrome: frequency and diagnosis. A lesson in conservatism. Clin Nephrol.

[13] Ricardo LGP, Zapata SI, Gonzalez RU (2018). Left inferior vena cava with nutcracker syndrome: a case report. Radiol Case Rep.

[14] Ha TS, Lee EJ (2006). ACE inhibition can improve orthostatic proteinuria associated with nutcracker syndrome. Pediatr Nephrol.

[15] Scholbach T (2007). From the nutcracker-phenomenon of the left renal vein to the midline congestion syndrome as a cause of migraine, headache, back and abdominal pain and functional disorders of pelvic organs. Med Hypotheses.

[16] Wang L, Yi L, Yang L, Liu Z, Rao J, Liu L, Yang J (2009). Diagnosis and surgical treatment of nutcracker syndrome: a single-center experience. Urology.

